# Parameterization of Four Models to Estimate Crop Evapotranspiration in a Solar Greenhouse

**DOI:** 10.3390/plants13111579

**Published:** 2024-06-06

**Authors:** Shikai Gao, Yu Li, Xuewen Gong, Yanbin Li

**Affiliations:** 1 School of Water Conservancy, North China University of Water Resources and Electric Power, Zhengzhou 450045, China; gaoshikai@ncwu.edu.cn (S.G.); x202210010036@stu.ncwu.edu.cn (Y.L.); liyanbin@ncwu.edu.cn (Y.L.); 2 Henan Key Laboratory of Crop Water Use, Zhengzhou 450045, China

**Keywords:** crop coefficient method, evapotranspiration, greenhouse tomato, Penman–Monteith, Priestley–Taylor, Shuttleworth–Wallace

## Abstract

Working to simplify mechanistic models on the basis of reliability for estimating crop evapotranspiration (ET) in a greenhouse is still worthwhile for horticulturists. In this study, four ET models (Penman–Monteith, Priestley–Taylor, and Shuttleworth–Wallace models, and the Crop coefficient method) were parameterized after taking the restriction effect of resistance parameters in these models on ET into account, named as PA-PM, PA-PT, PA-CC, and PA-SW, respectively. The performance of these four parameterized models was compared at different growth stages, as well as the entire growing season. Tomatoes that were ET-grown in a solar greenhouse without a heating device were measured using weighting lysimeters during 2016–2017 and 2019–2021, in which data from 2016 were used to adjust the model parameters, and data from the other four study years were used to examine the model performance. The results indicated that the PA-PT and PA-CC models have a better performance in estimating tomato ET at four growth stages, while the PA-PM and PA-SW performed well only at the development and middle stages. Compared to the ET that was measured with the weighting lysimeters, the ET that was predicted using the PA-PM model was 27.0% lower at the initial stage, and 8.7% higher at the late stage; the ET that was computed using the PA-SW model was 19.5% and 13.6% higher at the initial and late stages, respectively. The PA-PT model yielded the lowest root mean square error and the highest index of agreement against the other models over the entire growing season, indicating that the PA-PT model is the best recommended model for estimating tomato ET in a solar greenhouse.

## 1. Introduction

The rapid development of facility agriculture around the world provides high-quality vegetables, which greatly meet the demand for human beings for a quality life. In China, the greenhouse industry has created great economic value for local farmers while improving planting efficiency [1,2]. Greenhouse production, unlike field cultivation, requires rigorous and correct irrigation scheduling and climate conditions to maintain high production levels and crop quality [3]. Improving the reliability and precision of the irrigation amount and frequency for greenhouse crops over the entire growing season is a challenge for horticulturists and agriculturalists. Evapotranspiration (*ET*), as the outgoing vapor flux at the boundary between the soil, plant, and atmosphere, is a key variable for optimizing irrigation scheduling, and has been supported by many studies [3,4,5,6,7,8].

Measuring *ET*, or simulating it numerically, is a common way for mastering its pattern changes. In greenhouse climates with semi-enclosed structures, instruments based on field measurements require relatively complex physical principles and techniques, such as soil water balance [9,10,11,12], lysimeters [13,14,15], Bowen ratio [16], and sap flow plus micro-lysimeters [17,18]. In addition, instrumental measurement is expensive, time-consuming, and complex; further, it is difficult to avoid errors when using instrumental measurement [5,16,19,20]. Given the limitation of field measurements, therefore, obtaining *ET* by establishing a reliable mathematical model provides an effective alternative method that many studies give priority to [7].

At present, models frequently used in greenhouse for computing crop *ET* include the Penman–Monteith (PM), Priestley–Taylor (PT), and Shuttleworth–Wallace (SW) models, and the Crop coefficient method (CC). The single-layer standard PM model has been widely used for estimating *ET* in a variety of greenhouse types. The canopy cover is schematized as a single “big leaf” in the PM model and placed at a certain height within the canopy. The successful use of the PM model relies upon two key parameters, *e.g*., surface resistance (*r_s_*) and aerodynamic resistance (*r_a_*). The *r_s_* is a bulk resistance parameter that acts in series between the canopy surface and soil surface, while the *r_a_* can be defined as a function of the wind speed and canopy height [11]. However, the PM “single-layer” model faces the problem of application with simple and easily accessible parameters, especially for *r_s_* and *r_a_*. In the past few years, some empirical and semi-empirical models have been developed to calculate the *r_s_* by introducing terms or functions to make these models more suitable for special environmental conditions and improve the accuracy of *ET* estimation [21,22,23,24,25,26,27]. Among them, the Jarvis–Stewart model has been widely applied due to its physical representation of the environmental conditions controlling *ET* [4,13,14,28]. Another approach to estimating the *r_a_* parameter in greenhouses is the heat transfer coefficient approach [29], which can be used due to the low wind speed (<0.5 m/s at most of the days). However, calculating the heat transfer coefficient requires the accurate real-time acquisition of the canopy temperature, which has high requirements on measuring instruments, giving rise to uncertainties in the estimation of *r_a_*, especially under a sparse canopy space. In general, the complex computation process of *r_a_* and *r_s_* has slowed down the routine application of the PM model by farmers. Allen et al. [11] suggested that the canopy resistance can be considered as a constant, independent of climatic conditions; this simplifies the calculation procedure of the PM equation, but has only been verified on irrigated grass. Fernández et al. [30] introduced *r_a_* as a fixed value of 295 s/m into the PM model for computing crop *ET* in greenhouses. These results provide guidance for the parameterization of the PM model in greenhouses.

The PT model is a simplification of the Penman equation but it neglects the influence of vapor pressure deficit on crop *ET* [31]. The PT model assumes that *ET* is the product of the equilibrium evaporation and PT coefficient (*α_PT_*). The former depends only on solar radiation and air temperature, while the latter was an empirical parameter, influenced by the complexity of the natural environment [32,33]. Therefore, using the PT model to calculate *ET* in a greenhouse with low wind speed is convenient. Previous research reported that the successful use of the PT model depends on the accurate determination of *α_PT_* [34,35,36,37]. A well-known *α_PT_* was determined to be 1.26 by using daily measurements of *ET* on many vegetated areas, while subsequent studies and theories pointed to a near-constant *α_PT_* ∈ [1.2, 1.3] [31,34,38,39]. Conversely, other studies indicated that the value of the *α_PT_* had a large fluctuation over the whole growth period, and was mainly controlled by the mulching styles, leaf area index, air temperature, soil water content, relative humidity, etc. [35,40,41,42,43]. The improved *α_PT_* equation, which was developed by considering the above factors, generally has a higher simulation accuracy, but the simple PT model becomes more complicated due to the many parameters involved. Therefore, developing a simple *α_PT_* equation without losing the precision of the PT model in a greenhouse climate is still worthwhile work.

The crop coefficient method was a common method for computing *ET* since the publication of the FAO56 guidelines in 1998. In this method, crop *ET* was generated using the product of the specific crop coefficient (K_c_) and reference evapotranspiration (*ET_o_*). Here, *ET_o_* is the potential evaporating power of the atmosphere at a uniform underlying surface and can be calculated from weather data in reference crops, such as clipped grass or alfalfa [11,19]. Our previous study reported that taking the value of r_a_ as a constant of 308 s/m in a solar greenhouse was very close to the measurement [17]. The *K_c_* synthesizes physical and physiological differences between crop-specific surfaces and reference surfaces, and varies with management factors (e.g., crop type, climate, irrigation methods, etc.); hence, the *K_c_* plays an important role in practical applications [44]. The segmented curves of the *K_c_* were described in the FAO 56 guidelines, which is an approximate representation of the change in crop vegetation from planting to harvesting. At the initial and middle stages, the *K_c_* can be represented by two horizontal lines, since the variation in the crop growth in these two stages with time is small. The *K_c_* at the development and late stages is characterized by rising and falling line segments, respectively, which is closely related to the canopy cover of crop vegetables. The *K_c_* values for most crops were recommended in the FAO56 guidelines, which provided a convenient method for users [19]. Using the same *K_c_* values in different climates to estimate *ET*, however, was subject to significant uncertainties, as the *K_c_* varies greatly with the environment [45,46]. As a result, the *K_c_* needs to be amended based on local standards to guide irrigation scheduling more accurately. Therefore, investigating the variation patterns in the *K_c_* and modifying them according to special climate conditions, are important for developing *ET*-based irrigation scheduling.

The dual-layer SW model consists of two standard PM equations used to calculate soil evaporation and plant transpiration, respectively, and weighted by a set of coefficients representing the combination of soil and canopy resistance [47]. In the SW model, two surface resistances are employed to regulate the transfer of heat and mass from soil (*r^s^_s_*) and plants (*r^s^_c_*), and three aerodynamic resistances (*r^a^_a_*, *r^a^_c_*, *r^a^_s_*) are used to regulate fluxes to the atmosphere [24]. Although the SW model was widely applied to calculate *ET* for most crop types, it has a large number of parameters that are hard to parameterize, resulting in more cumbersome calculation procedures [48]. The performance of the SW model has been examined in solar greenhouses for tomatoes [13] and in Venlo-type greenhouses for cucumbers [49]; also, several resistance parameters of the SW model were analyzed over the whole growth stage. In addition, Zhao et al. [50] conducted a sensitivity analysis to uncertainties in input parameters and resistance for the SW model, and found that sensitivity of compute ET in the *r^a^_a_*, *r^a^_c_*, and *r^a^_s_* are unapparent, which provides a valuable reference for the parameterization of the SW model in greenhouses.

In conclusion, an effective approach for significantly improving the accuracy of *ET* estimation is to recalibrate the parameters in the model, which has been widely raised by many scholars. However, the greater number of parameters that need to be calibrated, the greater the risk of overfitting [48]. Therefore, parameterizing the PM, PT, CC, and SW models in solar greenhouses according to the characteristic of a semi-enclosed climate is still meaningful work, so that the precision is acceptable and model structure is simplified. The purpose of our study is (1) to parameterize the Penman–Monteith, Priestley–Taylor, and Shuttleworth–Wallace models, and the Crop coefficient method, for estimating crop *ET* in solar greenhouses in North China, and (2) to evaluate the performance of these models at different growth stages and the entire growth period.

## 2. Parameterization of Four Models

### 2.1. Penman–Monteith Model

The Penman–Monteith (PM) model which is used for calculating ET describes the canopy cover as a “big leaf” placed at a reference height above the crop; the structure diagrams of the PM model can be found in Allen et al. [11]. The formula is given as follows:

(1) $$\lambda ET=\frac{\Delta \left(R_{n}-G\right)+\rho_{a}c_{p}\left[(e_{s}-e_{a})/r_{a}\right]}{\Delta +\gamma \left(1+r_{s}/r_{a}\right)}$$

**Figure 1:**
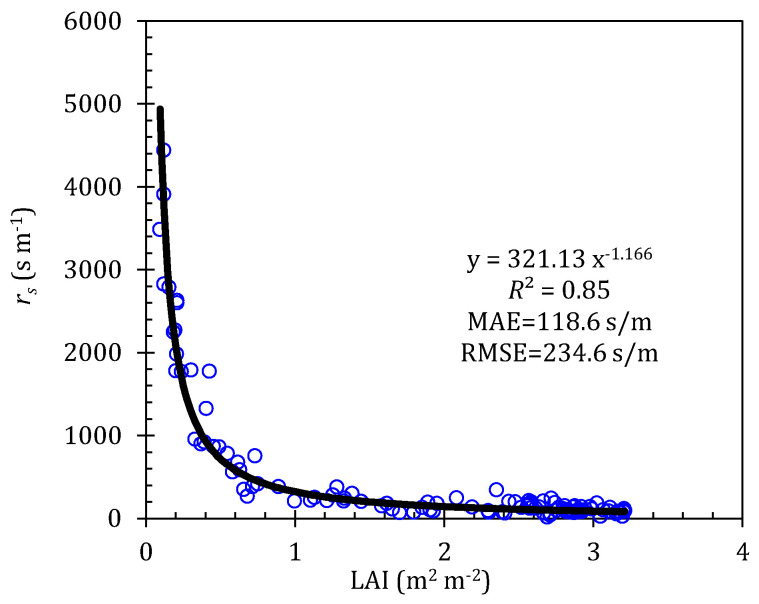
Relationship between surface resistance (*r_s_*) in Penman–Monteith model and leaf area index (LAI) by using 2016 study year. The black line is the fitting regression line.

In the PM model, physically based parameters of aerodynamic resistance (*r_a_*) and surface resistance (*r_s_*) are two key factors that affect the accuracy of the PM model. Three approaches for calculating *r_a_* were compared and evaluated in our previous study [17]; in this work, the *r_a_* variations were analyzed over the whole growth stage. To our surprise, setting the *r_a_* as a constant of 308 s/m is acceptable to calculate the ET. So, the measured *r_s_* can be calculated by inverting Equation (1) with the measured ET from three weighing lysimeters, and by taking *r_a_* as 308 s/m in this study. To simplify the calculation method of the *r_s_*, the statistical analysis revealed that a power function can be fitted for the relationship between the *r_s_* and LAI (see Figure 1).

### 2.2. Priestley–Taylor Model

The Priestley–Taylor (PT) model, based on equilibrium evaporation, is a simplified form of the Penman model [31]; the formula is expressed as follows:

(2) $$\lambda ET=\alpha_{PT}\cdot \lambda ET_{eq}=\alpha_{PT}\cdot \frac{\Delta }{\Delta +\gamma }\left(R_{n}-G\right)$$

**Figure 2:**
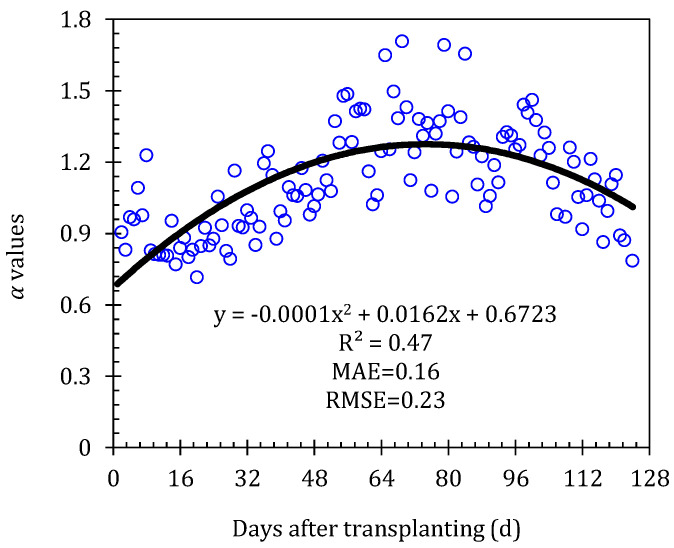
Relationship between Priestley–Taylor coefficient (α) and days after transplanting (DAT). Here α values were calculated by inverting the Priestley–Taylor model by using the 2016 *ET* data. The black line is the fitting regression line.

The successful use of the PT model lies primarily in determining the PT coefficient (*α_PT_*) [34,35], although some studies have shown that the coefficient *α_PT_* could be used as a constant (1.26) on some vegetated surfaces [33,36]. Conversely, many studies believe that the *α_PT_* was variable over the whole growing stage [34,42]. In this study, the coefficient *α_PT_* is computed by re-arranging the PT model, where the data of *λET* is from the weighing lysimeters. Thereafter, the relationship between the *α_PT_* and the days after transplanting (DAT) was established by using the data from the 2016 study years (see Figure 2).

### 2.3. Crop Coefficient Method

In the FAO56 manual, the crop coefficients (*K_c_*) can be computed by the ratio of the measured *ET* to the reference evapotranspiration (*ET_o_*).

(3) $$K_{c}=\frac{ET}{ET_{o}}$$

The *ET_o_* was calculated by using the FAO 56 Penman–Monteith formula with a fixed *r_a_* of 308 s/m [17], and is given as follows:

(4) $$ET_{o}=\frac{0.408\Delta \left(R_{n}-G\right)+\gamma \left[608(e_{s}-e_{a})/(T_{a}+273)\right]}{\Delta +1.23\gamma }$$

**Figure 3:**
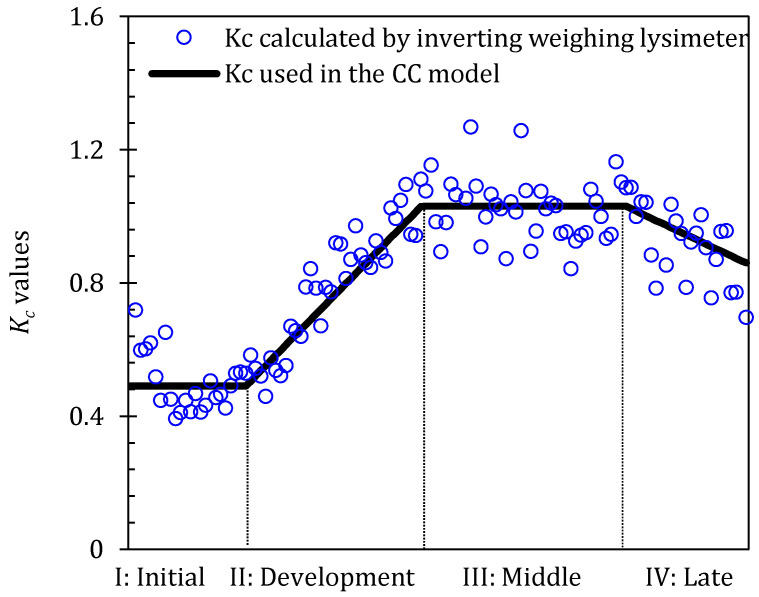
Variations of crop coefficient (*K_c_*) at four growth stages in 2016 study year. Here blue circles ο are *K_c_* values estimated by inverting the crop coefficient model using the 2016 *ET* data. The solid black line is the average value of *K_c_* at different growth stages.

Four growth stages (initial, development, middle, and late) were divided according to FAO Irrigation and Drainage Paper No. 56; the initial stage runs from the planting date to approximately a 10% ground cover, the development stage runs from a 10% ground cover to an effective full cover, the middle stage runs from the effective full cover to the start of maturity, and the late stage runs from the start of maturity to the harvest [11]. The observed length of the growth seasons for the greenhouse tomato is given in Table 1. The *K_c_* is defined as *K_cini_*, *K_cmid_*_,_ and *K_cend_* at the initial, middle, and late stages, respectively. Furthermore, the values of *K_cini_*, *K_cmid_*_,_ and *K_cend_* in the 2016 study year were obtained by using Equation (3) as 0.49, 1.03, and 0.86 (Figure 3 shows each respective segment of the linear relationship).

### 2.4. Shuttleworth–Wallace Model

In the Shuttleworth–Wallace (SW) model, soil evaporation and plant transpiration as one-dimensional models were combined. The surface resistance involved occurs at the interfaces between the soil and canopy and is further regulated by the aerodynamic resistance, which governs the transfer of water and heat between the soil and plants, plants and canopy, as well as soil and canopy; the structure diagrams of the SW model can be found in [47].

(5) $$\lambda ET=\lambda E+\lambda T=C_{s}PM_{s}+C_{c}PM_{c}$$

(6) $$PM_{s}=\frac{\Delta A+\left\{\left[\rho_{a}c_{p}VPD-\Delta r_{a}^{s}(A-A_{s})]/(r_{a}^{a}+r_{a}^{s}\right)\right\}}{\Delta +\gamma [1+r_{s}^{s}/(r_{a}^{a}+r_{a}^{s})]}$$

(7) $$PM_{c}=\frac{\Delta A+[\rho_{a}c_{p}VPD-\Delta r_{a}^{c}A_{s}]/(r_{a}^{a}+r_{a}^{c})}{\Delta +\gamma [1+r_{s}^{c}/(r_{a}^{a}+r_{a}^{c})]}$$

(8) $$C_{S}={\left[1+R_{s}R_{a}/R_{c}(R_{s}+R_{a})\right]}^{-1}$$

(9) $$C_{C}={\left[1+R_{c}R_{a}/R_{s}(R_{c}+R_{a})\right]}^{-1}$$

(10) $$R_{a}=(\Delta +\gamma )r_{a}^{a}$$

(11) $$R_{s}=(\Delta +\gamma )r_{a}^{s}+\gamma r_{s}^{s}$$

(12) $$R_{c}=(\Delta +\gamma )r_{a}^{c}+\gamma r_{s}^{c}$$

The meaning of the letters in the above equations can be found in the list of symbols and in Shutleworth and Wallace’s paper [47]. *A* and *A_s_* are given as follows:

(13) $$A=R_{n}-G$$

(14) $$A_{s}=R_{n}^{s}-G$$

(15) $$R_{n}^{s}=R_{n}\exp(-CLAI)$$

The extinction coefficient of light attenuation (*C*) was monitored by using two radiation sensors; one was placed 30 cm above the plant canopy and the other was placed on the soil surface. Based on the data from the 2016 study year, we found that the maximum value of *C* was 0.85 when the LAI reached its peak, and a value of 0 was taken for bare soil [13]. Thereafter, linear equations between *C* and LAI were used to compute *C* on each day.

**Figure 4:**
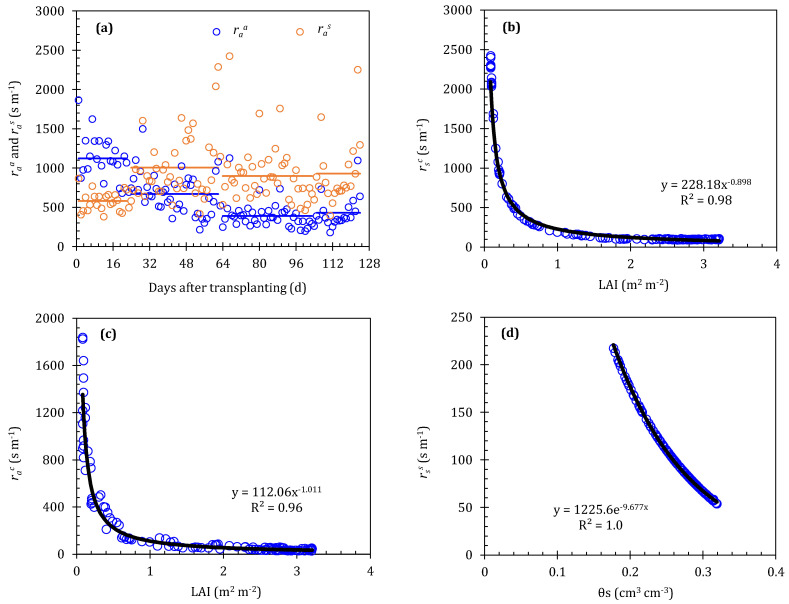
Resistance parameters in the Shuttleworth–Wallace model and its relationship with the influence factors. (**a**) Variations in *r_a_^a^* and *r_a_^s^* during the growing period, (**b**) relationship between *r_s_^c^* and LAI, (**c**) relationship between *r_a_^c^* and LAI, (**d**) and relationship between *r_s_^s^* and *θ_s_* in 2016 study year. The black line is the fitting regression line.

In the SW model, five resistances should be precisely calculated, including two aerodynamic resistances (*r*_a_^a^ and *r*_a_^s^), two surface resistance (*r*_s_^c^ and *r*_s_^s^), and one boundary layer resistance (*r*_a_^c^); the detailed calculations can be found elsewhere. The SW model improvement in this study is mainly developed to consider how to deal with these resistance parameters. Zhao et al. [50] indicated that the fluctuation of *r*_a_^a^ and *r*_a_^s^ was particularly insensitive to the SW model; our study attempts to define them as constants at different growth stages. The values of *r*_a_^a^ and *r*_a_^s^ in 2016 can be taken as 1124 and 576, 670 and 1005, 390 and 900, and 430 and 930 s/m at the initial, development, middle, and late stages, respectively (Figure 4a). So, these values were also used in other study years. To simplify the method of obtaining *r*_s_^c^ and *r*_s_^s^, we tested the relationship between *r*_s_^c^, *r*_s_^s^, and environmental factors in the 2016 study year, and found that a power function can be effectively fitted for the relationship between *r*_s_^c^ and LAI (*y* = 228.18*x*^−0.898^, *R*^2^ = 0.98) (Figure 4b), while an exponential function can be effectively fitted for the relationship between *r*_s_^s^ and θ_s_ (*y* = 1225.6e^−9.677*x*^, *R*^2^ = 1.0) (Figure 4d). These two relationships are also applied to other study years. The *r*_a_^c^ was also insensitive to the SW model; however, we found that *r*_a_^c^ and LAI have a good power function relationship (*y* = 112.06*x*^−1.011^, *R*^2^ = 0.96) (Figure 4c).

These parameters required by the Penman–Monteith, Priestley–Taylor, and Shuttleworth–Wallace models, and the Crop coefficient method, after parameterization are shown in Table 2.

### 2.5. Evaluation of Model Performance

Ten indexes were utilized to assess the performance of these improved models, e.g., the regression coefficient (*b*_0_), determination coefficient (*R*^2^), mean absolute error (*MAE*), root mean square error (*RSME*), rank sum ratio (*RSR*), average absolute error (*AAE*), average relative error (*ARE*), percent bias (*PBIAS*), modeling efficiency (*EF*), and index of agreement (*d_IA_*). Their formulas are given as follows [13,51]:

(16) $$b_{0}=\left[\sum_{i=1}^{n}O_{i}P_{i}/\sum_{i=1}^{n}O_{i}^{2}\right]$$

(17) $$R^{2}={\left[\frac{\sum_{i=1}^{n}\left(O_{i}-\bar{O}\right)\left(P_{i}-\bar{P}\right)}{\sqrt{\sum_{i=1}^{n}{\left(O_{i}-\bar{O}\right)}^{2}}\sqrt{\sum_{i=1}^{n}{\left(P_{i}-\bar{P}\right)}^{2}}}\right]}^{2}$$

(18) $$MAE=\frac{1}{n}\sum_{i=1}^{n}\left|O_{i}-P\right|$$

(19) $$RMSE={\left[\frac{\sum_{i=1}^{n}{\left(O_{i}-P_{i}\right)}^{2}}{n}\right]}^{0.5}$$

(20) $$RSR=\frac{{\left[\sum_{i=1}^{n}{\left(O_{i}-P_{i}\right)}^{2}\right]}^{0.5}}{{\left[\sum_{i=1}^{n}{\left(O_{i}-\bar{P}\right)}^{2}\right]}^{0.5}}$$

(21) $$AAE=\frac{1}{n}\sum_{i=1}^{n}\left|O_{i}-P_{i}\right|$$

(22) $$ARE=\frac{100}{n}\sum_{i=1}^{n}\left|\frac{O_{i}-P_{i}}{O_{i}}\right|$$

(23) $$PBIAS=100\frac{\sum_{i=1}^{n}\left(O_{i}-P_{i}\right)}{\sum_{i=1}^{n}O_{i}}$$

(24) $$EF=1.0-\frac{\sum_{i=1}^{n}{\left(O_{i}-P_{i}\right)}^{2}}{\sum_{i=1}^{n}{\left(O_{i}-\bar{O}\right)}^{2}}$$

(25) $$d_{IA}=1.0-\frac{\sum_{i=1}^{n}{\left(O_{i}-P_{i}\right)}^{2}}{\sum_{i=1}^{n}{\left(\left|P_{i}-\bar{O}\right|+\left|O_{i}-\bar{O}\right|\right)}^{2}}$$

where *O_i_* and *P_i_* (i = 1, 2, …, n) are the observed and predicted values, and $\bar{O}$ and $\bar{P}$ represent the mean values, respectively. The *MAE* can be used to reflect the actual prediction error. The *RMSE* is the variance in the estimated error. The *RSR* can be used to compare the mean, median, variance, and other indicators of two or more samples. The *AAE* can express the mean size of the estimation error. The size of the error in relative terms can be expressed by the *ARE*. The *PBIAS* can express the average tendency of the predicted values to their corresponding observations. The ratio of the mean square error to the variance in the observed data can be defined as the *EF*. The *d_IA_* can be used to represent the largest relative value that can occur from each observation-model simulation pair of values. In these indexes, the *b*_0_, *R*^2^, *EF*, and *d_IA_* are close to 1, while the *MAE*, *RMSE*, *RSR*, *AAE*, and *ARE* are close to 0, indicating that the improved model performs better.

All of the figures, tables, and data processing in this paper are completed by using the software of Microsoft Excel 2016.

## 3. Materials and Methods

### 3.1. Experiment Site and Description

The experiment was conducted from March to July 2016–2017 and 2019–2021 at the Xinxiang Comprehensive Experimental Base of the Chinese Academy of Agricultural Sciences located in Henan Province (35°86′ N, 113°68′ E, altitude 78.7 m). This site had a temperate continental climate with an average annual temperature of 14.2 °C, of which July was the hottest month, with a mean temperature of 27.1 °C, while January was the coldest month, with a mean temperature of 0.7 °C, and an average annual humidity of 68%. The average annual rainfall was 548 mm and the annual evaporation was 1908 mm. The most precipitation occurred from June to September, with a total rainfall of 409.7 mm, accounting for 72% of the annual precipitation.

A local steel-framed solar greenhouse was employed to carry out this study, which was topped with a 0.02 mm thick polyethylene film and fitted with vents (20 m in length × 0.5 m in width), with brick walls on the north, east, and west sides. The greenhouse was located in the east–west direction and covered an area of 510 m^2^. Tomato seedlings with varieties of *Solanum lycopersicum* and *c.v*. *Jinding* were transplanted into the greenhouse in early March, with a planting density of 5.7 plants per square meter, and a plant spacing of 30 cm; the division of the tomato growth stages can be found in Table 1. Irrigation events were controlled by referencing the 20 cm standard evaporation pan; irrigation was performed when the accumulated pan evaporation reached 20 ± 2 mm. In our study, drip irrigation was employed with a discharge rate of 1.1 L/h, and the irrigation amount was controlled by water meters.

### 3.2. Measurements

Three categories of content were measured, including meteorological factors, crop growth parameters, and soil characteristics.

Meteorological factors in the central area of the greenhouse, 2 m above the ground, were monitored by using an automatic weather station, including air temperature (*T_a_*), relative humidity (*RH*), net radiation (*R_n_*), canopy temperature (*T_c_*), soil heat flux (*G*), and wind speed (*u*_2_). A CS215 (Campbell Scientifc Inc., Logan, UT, USA) combined air temperature and relative humidity sensor was used for measuring T_a_ and RH. R_n_ was monitored by using an NR LITE2 net radiometer (Kipp & Zonen, Delft, The Netherlands), with a sensitivity of 10 μV/(W·m^2^). The factor of T_c_ was measured by using an SI-111 infrared radiation pyrometer (Campbell Scientifc Inc., Logan, UT, USA), installed 0.3 m above the canopy surface. Below 5 cm of the soil surface, two HFP01 soil heat flux plates (Hukseflux, Delft, The Netherlands) were employed to measure and adjust the G [40]. In addition, a high-precision air velocity meter (Wind Sonic, Gill, Nottingham, UK) with a precision of ±0.02 m/s was used to measure u_2_ inside the greenhouse. All meteorological data were collected in the data logger (Campbell Scientifc Inc., Logan, UT, USA) every 30 min.

The plant height and leaf area were measured manually at 7 d intervals; ten replications were set in this study. The missing data were obtained by using the ‘piecewise cubic Hermite interpolation polynomial” method in MATLAB 2020 software. The leaf area index (LAI) can be defined as the ratio of the plant leaf area to the unit soil surface area, which was determined by summing the rectangular area (leaf length × maximum width) of each leaf multiplied by a reduction factor of 0.64 [6].

The soil water content on the surface (0–5 cm) was continuously monitored using five ECH2O sensors (5TE, Decagon Devices, Inc., Pullman, WA, USA), which were distributed 15 cm away from the drippers along the line. The data were collected at 30 min intervals using an EM50 data logger (Decagon Devices, Inc., Pullman, WA, USA).

Crop evapotranspiration (*ET*) was monitored by using three weighing lysimeters, located in the middle of the greenhouse, with the specification of 1.0 m in length × 1.0 m in width × 1.2 m in depth. The lysimeter was filled from top to bottom with field soil for the first 80 cm depth, followed by a 10 cm thick fine aggregate and a 10 cm thick coarse aggregate to facilitate percolation. Six tomato plants were transplanted in each lysimeter with the same planting pattern as the field; bamboo poles were used to support plants to avoid lodging. The data of *ET* were reported using a micro-computer at 1.0 h intervals.

## 4. Results

### 4.1. Variations of Daytime Meteorological Factors, Leaf Area Index, and Soil Moisture in Five Study Years

**Figure 5:**
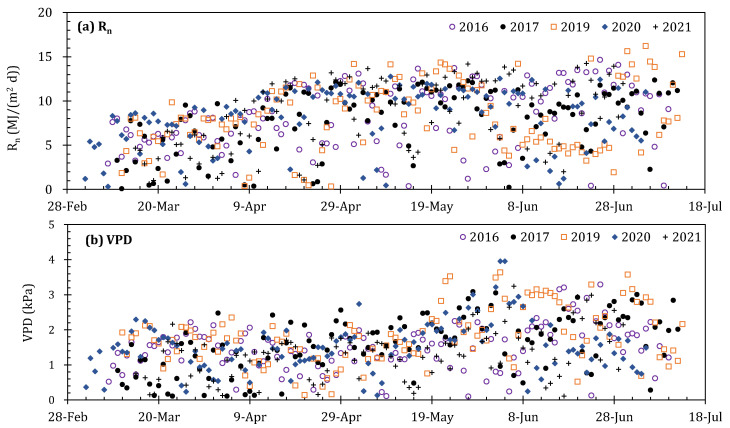

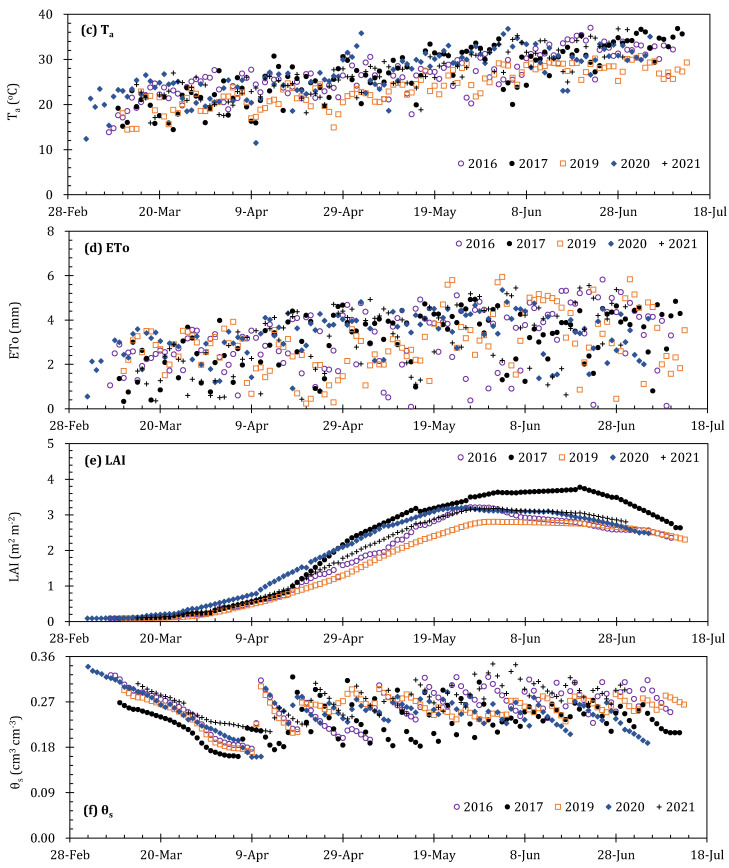
Variation in the daytime net radiation (*R_n_*, (**a**)), vapor pressure deficit (*VPD*, (**b**)), air temperature (*T_a_*, (**c**)), reference evapotranspiration (*ET_o_*, (**d**)), leaf area index (LAI, (**e**)), and surface soil volumetric water content (*θ_s_*, (**f**)) of greenhouse tomatoes during 2016–2017 and 2019–2021. Only the data ranged from 8:00 to 17:00 were adopted.

The variation patterns of meteorological factors (*R_n_*, *VPD*, *T_a_*), reference evapotranspiration (*ET_o_*), LAI, and surface soil water content (*θ_s_*) during the whole growth period in 2016–2017 and 2019–2021 were investigated primarily. Figure 5a shows the variation in the R_n_ in the daytime during the five study years. The daytime R_n_ ranged from 0.1 to 16.2 MJ/(m^2^·d), with an average of 8.2 MJ/(m^2^·d), and the maximum R_n_ often occurred on sunny days in June or July, while minimum values were frequently observed in overcast or rain days in every year. Figure 5b depicts that the daytime *VPD* varied from 0.1 to 4.0 kPa with an average of 1.6 kPa for the five study years. The variations in *VPD* in the greenhouse seem to be irregular, which is possibly due to the frequent occurrence of a high *T_a_* and high *RH* inside the greenhouse. The variations in the *T_a_* were consistent with the *R_n_*, which increases as the growth period progresses. The *T_a_* during the five years was between 11.5 and 37.0 °C with the average of daytime *T_a_* at seedling, development, middle, and late stages of 20.9, 23.8, 28.8, and 31.7 °C, respectively (Figure 5c).

The variations in the *ET_o_* showed a very similar trend with the *R_n_*, which fluctuated from 0.1 to 5.9 mm with a mean of 3.1 kPa. Because *R_n_* was the main control factor of the *ET_o_*. The average of the *ET_o_* at the seedling, development, middle, and late stages was 2.3, 2.9, 3.6, and 3.6 mm, respectively (Figure 5d). The tomato LAI presented a regular parabolic curve, and the LAI at the initial stage was relatively low, generally below 0.5 m^2^/m^2^. The maximum value of the LAI for every year differed remarkably (Figure 5e), with a maximum value of 3.2, 3.8, 2.8, 3.2, and 3.2 m^2^/m^2^ in the five years. The difference among the five years was mainly caused by crop growth and development. The variations in the *θ_s_* are displayed in Figure 5f; it varied from 0.17 to 0.35. Before the development stage, the soil moisture is relatively low, which is related to local cultivation practices. Generally, 20 mm of irrigation water was injected after transplantation; then, the tomato seedlings are squatted until they enter the development stage. Thereafter, irrigation events were performed again.

### 4.2. Performance of Parameterized Models for Simulating Daily ET at Four Growth Stages

**Figure 6:**
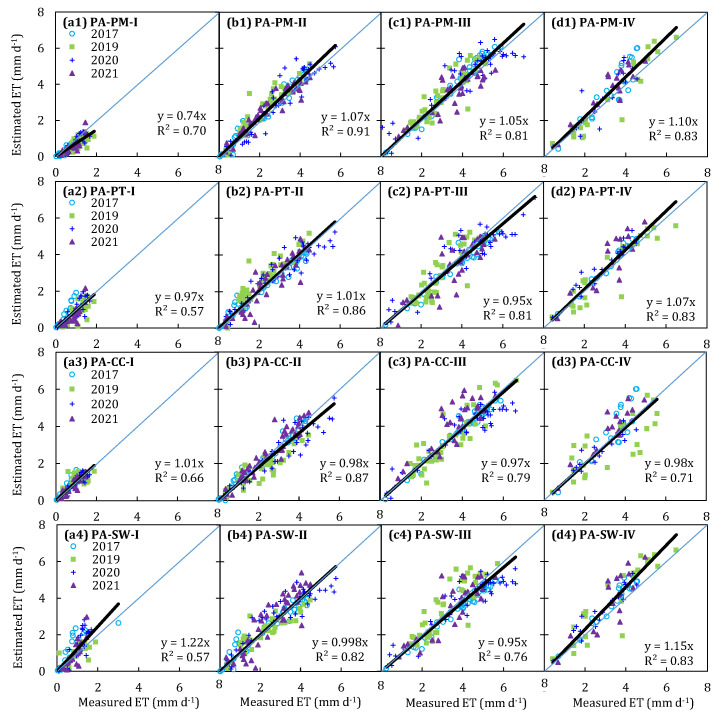
Comparison of daily *ET* estimated by the parameterized Penman–Monteith model (ET_PA-PM_, (**a1**–**d1**)), Priestley–Taylor model (ET_PA-PT_, (**a2**–**d2**)), crop coefficient method (ET_PA-CC_, (**a3**–**d3**)), Shuttleworth–Wallace model (ET _PA-SW_, (**a4**–**d4**)), and that measured by weighting lysimeters (ET_WL_) at four growth stages in 2017 and 2019–2021. The black line is the linear fitting regression line, and the blue line is the 1:1 line.

The performance of the parameterized Penman–Monteith (PA-PM), Priestley–Taylor (PA-PT), Shuttleworth–Wallace (PA-SW) models, and the Crop coefficient method (PA-CC) for estimating greenhouse tomato *ET* was evaluated. Table 3, Table 4, Table 5 and Table 6 and Figure 6 show the comparison of daytime *ET* estimated using the four parameterized models and measured using the weighting lysimeters at the initial, development, middle, and late stages in 2017 and 2019–2021.

At the initial stage, the PA-PM model presented a trend of underestimating the daily *ET*, whereas daily *ET* values were averagely overestimated by the PA-SW model in four study years. The statistical analysis shows that the PA-PM model seriously underestimated the *ET* by 27.0% (Figure 6(a1)), with a *MAE* of 0.27 mm/d, *RMSE* of 0.35 mm/d, and a positive *PBIAS* occurring in the PA-PM model (Table 3). However, the PA-SW model significantly overestimated the *ET* by 19.5% (Figure 6(a4)), with a *MAE* of 0.39 mm/d, *RMSE* of 0.56 mm/d, and a negative *PBIAS* occurring in the PA-SW model (Table 6). The PA-PT and PA-CC models show a better performance for estimating the daily ET at the initial stage; the b_0_ was close to 1.0, the *MAE* ranged from 0.21 to 0.29 mm/d, and the *RMSE* ranged from 0.28 to 0.38 mm/d in four years (Table 4 and Table 5).

At the development and middle stages, four parameterized models averagely produced the error of daily *ET*, ranging from 1.1% to 8.2% (Figure 6); these models yielded a *b*_0_ close to 1.0, and most of the *R*^2^ values were above 0.8, with the *MAE* changing from 0.39 to 0.57 mm/d, and the *RMSE* ranging from 0.51 to 0.71 mm/d. Furthermore, most of the *EF* values were near or above 0.8, and the *d_IA_* values were higher than 0.9. The statistical analysis also shows that the *RSR* values were lower than 0.5, and the *AAE* values were below 0.6 mm/d; the *ARE* varied from 16.5 to 31.5, and the *PBIAS* was within a positive and negative 10 value (Table 3, Table 4, Table 5 and Table 6).

At the late stage, the PA-PM and PA-SW models averagely overestimated the daily *ET* by 8.7% and 13.6%, respectively, while the daily *ET* estimated by PA-PT and PA-CC models was very close to the measurements in the four study years (Figure 6). The statistical analysis shows that the *MAE* and *RMSE* yielded by the PA-PT and PA-CC models were lower than 0.5 and 0.7 mm/d, whereas the *MAE* and *RMSE* produced by the PA-PM and PA-SW models were higher than 0.55 and 0.75 mm/d in 2017 and 2019–2021 (Table 3, Table 4, Table 5 and Table 6). In addition, compared with the PA-PM and PA-SW models, the PA-PT and PA-CC models have a lower *AAE*, *ARE*, and *PBIAS*, and a higher *EF* and *d_IA_*.

In summary, the precision of the PA-PM and PA-SW models in simulating the daily greenhouse tomato *ET* at the initial and late stages was unsatisfactory, while the PA-PT and PA-CC models showed a better performance. At the development and middle stages, the daily greenhouse tomato *ET* calculated by the four parameterized models agreed well with the measured *ET* from the weighting lysimeters. Among them, the PA-PT model was preferred in the greenhouse for estimating daily tomato *ET* due to its brief structure and fewer parameters.

### 4.3. Performance of Parameterized Models for Simulating Daily ET over the Whole Growth Stage

**Figure 7:**
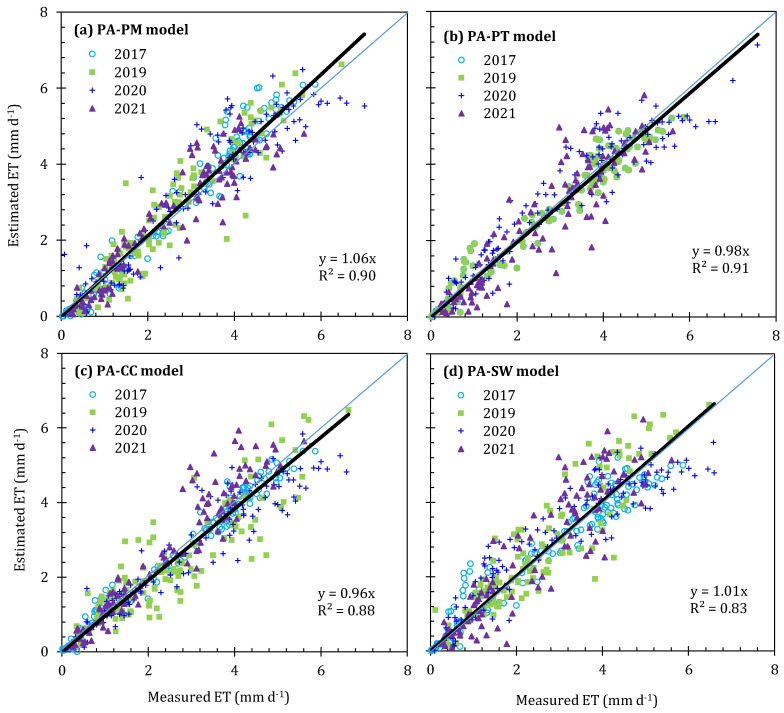
Comparison of daily *ET* estimated by the parameterized Penman–Monteith model (ET_PA-PM_, (**a**)), Priestley–Taylor model (ET_PA-PT_, (**b**)), crop coefficient method (ET_PA-CC_, (**c**)), Shuttleworth–Wallace model (ET_PA-SW_, (**d**)) and that measured by weighting lysimeter (ET_WL_) over the whole growth stage in 2017 and 2019–2021. The black line is the linear fitting regression line, and the blue line is the 1:1 line.

To evaluate the performance of four parameterized models for simulating daily *ET* systematically, 4 year weighting lysimeter data from 2017 and 2019–2021 were used to test the model precision over the whole growth stage (Table 7 and Figure 7). To our surprise, minor errors were found in all four parameterized models; the PA-PM and PA-SW models slightly overestimated the daily *ET* by 5.4% and 3.0%, respectively, while the daily *ET* was slightly underestimated by the PA-PT and PA-CC models, with values of 0.7% and 2.7% in the four study years (Figure 7). The statistical analysis shows that all models yielded the *R*^2^ higher than 0.8; particularly, the *R*^2^ of the PA-PT model was higher than 0.9 (Table 7). The *MAE* and *RMSE* produced from the PA-SW model were higher than other models, while the PA-PT model has the lowest *MAE* and *RMSE*, which were 0.38 and 0.51 mm/d (Table 7), indicating that the PA-PT model has the better performance among the four parameterized models. The indexes of the *RSR*, *AAE*, and *PBIAS* have a similar trend to the *MAE* and *RMSE*, indicating that the PA-PT model has a lower estimation error compared to other models. In addition, a smaller *ARE* was found in the PA-CC model (Table 7), indicating that the estimation error amplitude of the PA-CC model was small over the whole growth period, that is, the stability of the PA-CC model in greenhouses was better than the PA-PM and PA-SW models.

The consistency index evaluation of the four parameterized models shows that the EF and *d_IA_* yielded from PA-PT model were the highest, indicating that the mean square error of the observed data was very close to the variance, and a small gap in the maximum relative value of the simulation results was obtained by using the PA-PT model. Although the consistency index of the PA-PM, PA-CC, and PA-SW models were slightly lower compared with the PA-PT model, their performance was still satisfactory, with the *EF* and *d_IA_* above 0.8 and 0.95 (Table 7), respectively. In a word, the PA-PT model was recommended due to a high simulation accuracy and easier access to model parameters.

## 5. Discussion

### 5.1. Evaluation of Four Parameterized Models to Compute ET in Greenhouse

In our study, four models were parameterized to compute the *ET* of greenhouse-grown tomatoes, including the Penman–Monteith, Priestley–Taylor, and Shuttleworth–Wallace models, and the Crop coefficient method. The results indicated that the PA-PM and PA-SW models severely under- and overestimated the tomato *ET* at the initial stage, respectively, while they slightly overestimated the tomato *ET* at the late stage. The performance of the PA-PM and PA-SW models, however, was better at the development and middle stages (Figure 6 and Table 3 and Table 6). The PA-PT and PA-CC models performed well at the four growth stages. The performance of the PA-PM model at the initial stage was somewhat different from the results obtained by Palmer et al. [52]; these authors pointed out that the PM model tended to over-predict the *ET* when the LAI becomes small. Its imperfection is not surprising; the surface resistance of the PA-PM model in our study was fitted using the LAI as a power function, resulting in the fitted *r_s_* increasing rapidly with the decrease in the LAI (LAI < 0.5 m^2^/m^2^ at the initial stage). The larger the *r_s_*, the smaller the *ET* simulated by the PM model, which has been confirmed in many studies [4,14,17,53,54]. Conversely, the *r_s_* was underestimated at the late stage due to a higher LAI, which was because infinite types of tomato varieties were used in our study, resulting in less leaf aging at the late stage. But the performance of the PA-PM model was not greatly affected, as its overestimation was only 8.7% (Figure 6). During the development and middle periods, the LAI of tomatoes ranged from 1.0 to 3.5 m^2^/m^2^; the PA-PM model can effectively predict the tomato *ET*, indicating that calculating the r_s_ was feasible by using the LAI as a power function. So, the assumption that the underlying surface is treated as a “big leaf” in the PM model was the key to ensure its accuracy [7,14,22,33,48,52,54]. In addition, such results also illustrated that surface resistance played a critical role in the performance of the PA-PM model, especially during the sparse canopy stage.

The PA-SW model over-predicted the *ET* by 19.5% and 13.6% at the initial and late stages, respectively (Figure 6). These results may be due to the lower tomato LAI, the overestimation of available energy intercepted by the canopy in the PA-SW model, as well as the restriction of soil water stress on water transport. Referring to local agronomic practices, tomato seedlings transplanted in greenhouses were irrigated with only 20 mm water to enhance the expansion of root system, and no water was applied before entering the development stage; additionally, a small amount of water was supplied at the late stage to improve the tomato quality. Thus, soil water stress frequently occurred at the initial and late stages. A number of studies have also reported that crop *ET* will be over-predicted by the SW model under water stress and low LAI conditions [13,50,54,55,56,57,58]. The PA-SW model performs well during the development and middle stages, which have been confirmed in our previous work [13]. Additionally, the PA-SW model still has errors in estimating the tomato *ET*, which is related to the large number of parameters in the model. A large number of parameters may increase the uncertainty of the model [53]. Notwithstanding its limitation, the PA-SW model indeed provides a simple method to predict tomato *ET* in greenhouse where the LAI > 1.0 m^2^/m^2^ and without water stress.

The PA-PT and PA-CC models performed well at all four growth stages, and fewer parameters were embedded in these two models, among which two important coefficients, *α_PT_* and *K_c_*, determined the successful computation of tomato *ET* in the greenhouse. Herein, the *α_PT_* was obtained by inverting Equation (2) and was then fitted with days after transplanting; the *K_c_* was obtained by inverting Equation (3) and was calculated using the linear interpolation method. Both the *α_PT_* and *K_c_* were corrected using measured data from the 2016 study year. The PT model was a simplification based on the available energy penman method, in which the radiation factor was the main component [31]. Yan et al. [59] indicated that the latent heat flux was the primary component of the net radiation over the whole growing season in the greenhouse and was principally constrained by net radiation. Similar results were also found elsewhere [3,5,6,7,16,59,60,61]. So, the performance of the PA-PT model was conceivable. In our study, tomatoes were grown in the same greenhouse using a similar time, pattern, and management practice; thus, the *K_c_* fluctuated smoothly, although the greenhouse environment varies slightly in each year. Therefore, taking the *K_c_* as 0.49, 1.03, and 0.86 at the initial, middle, and late stages, respectively, was perfect for computing the tomato *ET* in the greenhouse. The values of *K_c_* in this study were lower than the values recommended by the FAO56 guidelines (0.6, 1.15, and 0.9), especially at the middle stage, which may be due to the higher humidity and lower air speed in the greenhouse. In addition, the walkway installed in the greenhouse reduces the canopy coverage, which was another reason for the decrease in the *K_c_*. Hanson and May [62] concluded that the *K_c_* of greenhouse tomatoes at the middle stage ranged from 0.99 to 1.08 based on the data from three study years. Qiu et al. [63] also reported that the tomato *K_c_* in the greenhouse at the mid-season mark was much lower than the values recommended by the FAO56 guidelines and fluctuated between 0.77 and 0.97.

The four parameterized models can effectively compute the daily *ET* of greenhouse-grown tomatoes over the entire growth stage, although slight over- or underestimates are inevitable, but the errors were within the allowable range (*MAE* ranged from 0.38 to 0.51 mm/d) (Table 7 and Figure 7). The successful use of the models was mainly attributed to the consideration of the integrated restriction effect of the greenhouse micro-environment, plant morphology, and soil moisture on the model parameters. Therefore, the parameterized method of the PM, PT, CC, and SW models to estimate the *ET* for greenhouse-grown tomatoes with drip irrigation was acceptable.

### 5.2. Advantages and Disadvantages of Four Parameterized Models

The PA-PT and PA-CC models can provide an accurate estimation of *ET* for tomatoes in greenhouses at different growth stages, especially at the initial and late stages with low coverage and water deficit. Additionally, the PA-PT and PA-CC models were simpler and easier to use than the PA-PM and PA-SW models, due to fewer parameters. Of course, some errors still occur when estimating *ET* using these two models. Firstly, the discrepancy of *R*^2^ and EF between ET estimated by the PA-PT model and measurements was larger than PA-PM and PA-CC models at the initial stage, which may be attribute to the lower available energy received by the canopy in sparse LAI stage. Secondly, the *ET_o_* should be confirmed accurately in the PA-CC model; however, the PM model fixed a constant *r_a_* of 308 s/m, which was acceptable. Therefore, putting forward the exact formula for calculating the *ET_o_* in a solar greenhouse was quite clearly necessary.

As for the PA-PM model, it provided an accurate *ET* estimate when the soil subsurface was fully covered; in this case, the underlying surface can be treated as a whole, completely satisfying the hypothesis of the “big leaf”. Additionally, the performance of the PA-PM model also decreases when soil water was limited [64]. Regrettably, the PA-PM model does not take into account the contribution of soil layers to evaporation; hence, it is not suitable for partial or sparse canopy conditions. Nyolei et al. [4] indicated that the application of the surface resistance model was not appropriate in the case where soil water vapor was the main source of the *ET*, especially during the early stages of crop development, as the approach underestimates the *ET* during this stage. Therefore, the LAI was used as a power function to calculate the *r_s_* at the initial stage, which may produce a great error.

The PA-SW model is probably the most cumbersome of these four parameterized models because many parameters should be adjusted before application, which may increase the uncertainty of the model. Even though five resistances in the PA-SW model were parameterized in our study, we still did not improve the accuracy when the LAI was below 1.0 m^2^/m^2^. Nyolei et al. [4] considered that developing a segmented method of surface resistance calibration can improve the performance of models to make them better adaptable to environmental changes. However, at present, it is still unclear how all environmental variables in greenhouses correlate with surface resistance, because the surface resistance was simultaneously affected by several environmental variables. Li et al. [28] also indicated that unsegmented models decrease the canopy resistance, thereby overestimating the *ET*. Notwithstanding its limitation, the PA-SW model does serve as a simple approach for users to choose in estimating the tomato *ET* under the dense canopy stage. Additionally, the PA-SW model can also be used to estimate soil evaporation and plant transpiration separately, which provides an effective tool for reducing “ineffective evaporation” from the soil layer.

In conclusion, our results can provide a simple method and theoretical basis for estimating crop *ET* in greenhouses, especially by parameterizing the Priestley–Taylor model, whose parameters are more readily available and more acceptable to farmers.

## 6. Conclusions

The application of mechanistic evapotranspiration models is often limited due to the large number of parameters and the need for calibration according to environmental variables. In this study, the Penman–Monteith, Priestley–Taylor, and Shuttleworth–Wallace models, and the crop coefficient method were parameterized, and their performance in the estimation of tomato *ET* in a greenhouse was estimated. The PA-PT model estimated the *ET* more accurately than the PA-PM, PA-CC, and PA-SW models, both at different stages and the entire growing season. Furthermore, the parameters in the PA-PT model were minimal and easier to obtain, thereby reducing the model uncertainty. The second recommended model was the PA-CC model; although the accuracy was slightly lower than the PA-PT model, its convenience may be more acceptable to farmers. The discrepancy was found between the *ET* estimated by the PA-PM and PA-SW models and measurements at the initial and late stages, where the underlying surface was sparse and crops frequently suffered from water stress. Considering the large number of parameters in the Shuttleworth–Wallace model, the PA-SW model provides a simple approach to predict crop *ET* in greenhouses, which is helpful for its practical applications. By comparing the structure and accuracy of these four parameterized models, the PA-PT model is recommended to estimate crop *ET* in a greenhouse. These parameterized models can be used in an easy and relatively accurate way for determining greenhouse tomato water consumption and developing appropriate irrigation scheduling.

## Figures and Tables

**Table 1:** Information of growing stages and irrigation evens for greenhouse tomato during 2016–2017 and 2019–2021.

Years	Growing Stages				Irrigation Number	Irrigation Amount (mm)
	Initial	Development	Middle	Late		
2016	9–31 March	1 April–10 May	11 May–20 June	21 June–10 July	15	282.7
2017	1 March–11 April	2 April–10 May	11 May–21 June	22 June–12 July	15	308.2
2019	12 Mar–2 April	3 April–11 May	12 May–20 June	21 June–13 July	15	311.7
2020	4–25 March	26 March–5 May	6 May–16 June	17 June–7 July	14	270.5
2021	6–15 March	7 April–16 May	17 May–14 June	15–30 June	14	274.4

**Table 2:** The different parameters required by four parameterized models.

Parameterized Models	Meteorological-Based Parameters	Crop Parameters	Soil Parameters
Penman–Monteith	*R_n_*, *G_s_*, *RH*, *T_a_*, *ρ*, *C_p_*, *γ*	LAI	/
Priestley–Taylor	*R_n_*, *G_s_*, *T_a_*, *γ*	DAT	/
Crop coefficient method	*R_n_*, *G_s_*, *RH*, *T_a_*, *γ*	DAT	/
Shuttleworth–Wallace	*R_n_*, *G_s_*, *RH*, *T_a_*, *ρ*, *C_p_*, *γ*	DAT, C, LAI	*θ_s_*

The meaning of the letters in Table 2 can be found in the list of symbols.

**Table 3:** Statistical results of daily *ET* estimated using the parameterized Penman–Monteith model at four growth stages in 2017 and 2019–2021.

Stages	Year	*b*_0_	*R* ^2^	*MAE*	*RMSE*	*RSR*	*AAE*	*ARE*	*PBIAS*	*EF*	*d_IA_*
Initial	2017	0.67	0.75	0.20	0.28	0.64	0.20	43.54	34.40	0.49	0.85
	2019	0.69	0.24	0.35	0.45	0.93	0.35	28.99	29.03	0.46	0.64
	2020	0.82	0.71	0.28	0.30	0.67	0.23	24.13	18.13	0.44	0.87
	2021	0.73	0.66	0.28	0.33	0.69	0.28	37.79	31.03	0.20	0.83
	**4 years**	0.74	0.70	0.27	0.35	0.68	0.27	33.66	27.04	0.40	0.85
Development	2017	1.07	0.97	0.26	0.35	0.19	0.26	22.40	−7.53	0.96	0.99
	2019	1.19	0.90	0.48	0.62	0.45	0.48	30.28	−19.73	0.78	0.96
	2020	1.05	0.87	0.48	0.63	0.35	0.48	21.76	−4.29	0.87	0.97
	2021	1.03	0.89	0.32	0.40	0.33	0.32	18.03	−6.01	0.90	0.98
	**4 years**	1.07	0.91	0.39	0.51	0.36	0.39	23.07	−8.64	0.86	0.97
Middle	2017	1.09	0.96	0.39	0.49	0.32	0.39	9.99	−8.78	0.87	0.97
	2019	1.10	0.80	0.50	0.66	0.58	0.50	17.44	−10.61	0.63	0.92
	2020	1.02	0.75	0.69	0.86	0.46	0.69	76.54	−5.53	0.79	0.94
	2021	0.98	0.64	0.54	0.67	0.58	0.54	16.64	0.36	0.69	0.92
	**4 years**	1.05	0.81	0.53	0.68	0.46	0.53	31.47	−6.65	0.79	0.95
Late	2017	1.14	0.79	0.66	0.82	0.77	0.66	19.43	−12.90	0.28	0.88
	2019	1.02	0.90	0.47	0.62	0.37	0.47	18.64	−1.20	0.87	0.97
	2020	1.13	0.78	0.59	0.82	0.69	0.59	21.69	−12.78	0.48	0.90
	2021	1.15	0.81	0.62	0.76	0.64	0.62	19.44	−16.45	0.49	0.89
	**4 years**	1.10	0.83	0.58	0.75	0.57	0.58	19.74	−9.55	0.66	0.93

**Table 4:** Statistical results of daily ET estimated using the parameterized Priestley–Taylor model at four growth stages in 2017 and 2019–2021.

Stages	Year	*b*_0_	*R* ^2^	*MAE*	*RMSE*	*RSR*	*AAE*	*ARE*	*PBIAS*	*EF*	*d_IA_*
Initial	2017	1.25	0.68	0.25	0.38	0.90	0.25	39.07	−25.36	0.08	0.85
	2019	0.83	0.35	0.33	0.43	1.05	0.33	31.34	16.13	0.41	0.72
	2020	1.09	0.61	0.33	0.39	0.97	0.33	33.38	−7.42	0.08	0.84
	2021	0.83	0.60	0.24	0.32	0.74	0.24	31.04	20.11	0.23	0.84
	**4 years**	0.97	0.57	0.29	0.38	0.85	0.29	33.68	3.40	0.28	0.85
Development	2017	0.95	0.96	0.23	0.32	0.17	0.57	18.32	2.16	0.97	0.99
	2019	1.18	0.88	0.48	0.63	0.54	0.48	30.05	−20.37	0.76	0.95
	2020	0.98	0.83	0.47	0.57	0.30	0.47	21.32	1.94	0.92	0.98
	2021	0.99	0.84	0.40	0.54	0.43	0.40	21.03	1.61	0.83	0.96
	**4 years**	1.01	0.86	0.40	0.53	0.37	0.40	22.65	−3.92	0.86	0.96
Middle	2017	0.93	0.95	0.34	0.40	0.31	0.33	9.86	7.29	0.91	0.98
	2019	1.01	0.74	0.51	0.66	0.64	0.51	17.84	−0.43	0.58	0.92
	2020	0.94	0.83	0.59	0.73	0.39	0.59	21.98	2.92	0.85	0.96
	2021	0.94	0.62	0.68	0.83	0.75	0.68	22.16	6.14	0.46	0.88
	**4 years**	0.95	0.81	0.51	0.67	0.45	0.51	17.60	4.28	0.79	0.95
Late	2017	1.08	0.97	0.28	0.33	0.34	0.28	8.05	−7.76	0.88	0.97
	2019	0.97	0.86	0.50	0.66	0.39	0.50	20.65	2.27	0.85	0.96
	2020	1.14	0.89	0.48	0.58	0.47	0.48	20.48	−15.73	0.74	0.94
	2021	1.13	0.74	0.73	0.86	0.75	0.73	23.46	−12.89	0.34	0.88
	**4 years**	1.07	0.83	0.48	0.62	0.48	0.48	17.83	−7.59	0.77	0.95

**Table 5:** Statistical results of daily ET estimated using the parameterized crop coefficient model at four growth stages in 2017 and 2019–2021.

Stages	Year	*b*_0_	*R* ^2^	*MAE*	*RMSE*	*RSR*	*AAE*	*ARE*	*PBIAS*	*EF*	*d_IA_*
Initial	2017	1.18	0.79	0.17	0.26	0.64	0.17	29.51	−20.06	0.56	0.91
	2019	1.00	0.35	0.32	0.39	1.05	0.32	34.14	−7.28	0.62	0.60
	2020	0.97	0.71	0.21	0.23	0.58	0.21	20.49	1.54	0.67	0.92
	2021	0.96	0.79	0.15	0.19	0.52	0.15	23.35	4.58	0.73	0.94
	**4 years**	1.01	0.66	0.21	0.28	0.62	0.21	26.83	−3.98	0.62	0.90
Development	2017	0.98	0.97	0.22	0.28	0.15	0.22	23.34	0.86	0.98	0.99
	2019	0.78	0.82	0.53	0.64	0.49	0.53	27.02	19.36	0.75	0.91
	2020	0.85	0.87	0.55	0.72	0.32	0.55	17.96	14.99	0.87	0.96
	2021	1.03	0.91	0.29	0.40	0.29	0.29	15.89	−3.12	0.91	0.98
	**4 years**	0.91	0.87	0.40	0.54	0.38	0.40	20.98	8.19	0.87	0.97
Middle	2017	0.96	0.96	0.24	0.29	0.22	0.24	8.19	4.03	0.95	0.99
	2019	0.98	0.80	0.52	0.66	0.51	0.52	17.89	1.91	0.74	0.94
	2020	0.90	0.83	0.60	0.75	0.41	0.60	19.73	7.05	0.82	0.95
	2021	1.12	0.65	0.68	0.88	0.65	0.68	21.82	−13.98	0.43	0.86
	**4 years**	0.97	0.79	0.50	0.66	0.46	0.50	16.48	1.09	0.79	0.94
Late	2017	0.99	0.97	0.14	0.99	0.19	0.14	3.84	1.64	0.96	0.99
	2019	0.88	0.57	0.81	1.02	0.64	0.81	28.78	8.11	0.59	0.88
	2020	0.96	0.87	0.30	0.39	0.34	0.30	11.80	2.30	0.88	0.97
	2021	1.15	0.85	0.54	0.71	0.60	0.54	17.15	−15.72	0.55	0.91
	**4 years**	0.98	0.71	0.46	0.67	0.53	0.46	15.71	0.13	0.71	0.92

**Table 6:** Statistical results of daily ET estimated using the parameterized Shuttleworth–Wallace model at four growth stages in 2017 and 2019–2021.

Stages	Year	*b*_0_	*R* ^2^	*MAE*	*RMSE*	*RSR*	*AAE*	*ARE*	*PBIAS*	*EF*	*d_IA_*
Initial	2017	1.34	0.61	0.43	0.63	0.85	0.43	60.10	−54.60	0.04	0.82
	2019	0.98	0.30	0.26	0.36	0.85	0.26	57.02	2.17	0.28	0.80
	2020	1.42	0.61	0.59	0.72	1.29	0.59	57.02	−38.53	−2.17	0.70
	2021	1.19	0.63	0.28	0.46	1.24	0.28	31.10	−10.42	−0.53	0.81
	**4 years**	1.22	0.57	0.39	0.56	1.01	0.39	51.07	−24.26	−0.21	0.80
Development	2017	0.97	0.92	0.28	0.40	0.23	0.28	24.65	0.37	0.96	0.99
	2019	0.93	0.77	0.38	0.49	0.40	0.38	22.43	1.06	0.86	0.96
	2020	0.98	0.77	0.53	0.64	0.35	0.53	24.83	2.81	0.90	0.97
	2021	1.11	0.81	0.62	0.73	0.61	0.62	31.50	−9.40	0.68	0.94
	**4 years**	1.00	0.82	0.46	0.58	0.41	0.46	25.87	−3.10	0.87	0.96
Middle	2017	0.89	0.95	0.45	0.50	0.39	0.45	12.79	10.65	0.85	0.97
	2019	1.09	0.76	0.58	0.76	0.65	0.58	19.22	−8.17	0.55	0.91
	2020	0.90	0.81	0.66	0.81	0.44	0.66	21.72	7.87	0.80	0.94
	2021	1.00	0.71	0.58	0.76	0.61	0.58	18.17	−0.14	0.63	0.92
	**4 years**	0.95	0.76	0.57	0.71	0.49	0.57	17.94	3.68	0.76	0.93
Late	2017	1.13	0.93	0.45	0.55	0.54	0.45	13.03	−12.74	0.68	0.94
	2019	1.14	0.83	0.80	0.97	0.56	0.80	29.33	−15.55	0.67	0.93
	2020	1.15	0.89	0.51	0.61	0.49	0.51	21.62	−16.80	0.71	0.93
	2021	1.20	0.72	0.92	1.09	0.88	0.92	28.94	−18.99	0.41	0.83
	**4 years**	1.15	0.83	0.66	0.83	0.60	0.66	23.06	−15.73	0.58	0.91

**Table 7:** Statistical results of daily *ET* estimated using the parameterized Penman–Monteith model (PA-PM), Priestley–Taylor model (PA-PT), crop coefficient model (PA-CC), Shuttleworth–Wallace model (PA-SW) over the whole growth stage in 2017 and 2019–2021.

Models	Year	*b*_0_	*R* ^2^	*MAE*	*RMSE*	*RSR*	*AAE*	*ARE*	*PBIAS*	*EF*	*d_IA_*
PA-PM	2017	1.09	0.96	0.36	0.50	0.29	0.36	21.44	−7.73	0.92	0.98
	2019	1.08	0.88	0.46	0.61	0.44	0.46	23.75	−6.81	0.80	0.96
	2020	1.04	0.87	0.53	0.71	0.39	0.53	40.72	−5.00	0.85	0.96
	2021	1.03	0.89	0.42	0.54	0.36	0.42	22.08	−3.12	0.87	0.97
	**4 years**	1.06	0.90	0.44	0.59	0.36	0.44	27.16	−5.74	0.87	0.97
PA-PT	2017	0.97	0.95	0.28	0.36	0.21	0.28	19.54	1.72	0.95	0.99
	2019	0.97	0.95	0.28	0.36	0.21	0.28	18.10	1.77	0.95	0.99
	2020	0.98	0.87	0.49	0.68	0.47	0.49	23.56	0.17	0.78	0.95
	2021	0.99	0.84	0.49	0.66	0.45	0.49	23.82	1.80	0.80	0.95
	**4 years**	0.98	0.91	0.38	0.51	0.30	0.38	21.18	0.69	0.91	0.98
PA-CC	2017	0.97	0.98	0.20	0.27	0.16	0.20	16.00	1.79	0.98	0.99
	2019	0.91	0.79	0.54	0.70	0.48	0.54	25.67	7.19	0.77	0.94
	2020	0.89	0.89	0.47	0.63	0.35	0.47	18.07	8.65	0.87	0.96
	2021	1.10	0.91	0.40	0.59	0.40	0.40	19.26	−9.27	0.84	0.96
	**4 years**	0.96	0.88	0.41	0.57	0.35	0.41	19.77	2.73	0.88	0.97
PA-SW	2017	0.97	0.90	0.40	0.51	0.30	0.40	25.17	0.08	0.91	0.98
	2019	1.07	0.85	0.50	0.67	0.47	0.50	28.93	−7.23	0.78	0.95
	2020	0.96	0.79	0.58	0.71	0.40	0.58	28.56	2.44	0.84	0.95
	2021	1.08	0.83	0.58	0.76	0.51	0.58	27.46	−7.80	0.74	0.94
	**4 years**	1.01	0.83	0.51	0.67	0.41	0.51	27.54	−4.03	0.83	0.96

## Data Availability

Data are contained within the article.

## Nomenclature

*List of symbols*	
*A*	Total available energy (W/m^2^)
*A_s_*	Available energy to the soil surface (W/m^2^)
*C*	Extinction coefficient of light attenuation
*C_s_*	Soil surface resistance coefficient
*C_c_*	Canopy surface resistance coefficient
*C_p_*	Specific heat of dry air at a constant pressure (J/(kg·K))
*ET*	Evapotranspiration (mm)
*ET_o_*	Reference evapotranspiration (mm)
*e_s_*	Saturation vapor pressure (kPa)
*e_a_*	Actual vapor pressure (kPa)
*G*	Surface soil heat flux (W/m^2^)
LAI	Leaf area index
*K_c_*	Crop efficient
*R_n_*	Net radiation (W/m^2^)
*R_n_^s^*	Net radiation reaching the soil surface (W/m^2^)
*r_a_*	Aerodynamic resistance (s/m)
*r_s_*	Surface resistance (s/m)
*r_a_^a^*	Aerodynamic resistances from reference level to canopy source height (s/m)
*r_a_^c^*	Boundary layer resistance of the crop in the canopy (s/m)
*r_a_^s^*	Aerodynamic resistances from canopy source height to the soil surface (s/m)
*r_s_^c^*	Canopy surface resistance (s/m)
*r_s_^s^*	Soil surface resistance (s/m)
*VPD*	Vapor pressure deficit (s/m)
*α_PT_*	Priestley–Taylor coefficient
*ρ*	Air density (kg/m^3^)
*γ*	Psychometric constant (kPa/K)
Δ	Slope of the saturation water vapor pressure versus temperature curve (kPa/°C)
